# Muting the self”: emotional dysregulation & appeareance and performance-enhancing drugs (APEDs); a clinical report

**DOI:** 10.1192/j.eurpsy.2025.1201

**Published:** 2025-08-26

**Authors:** G. García Cepero, M. E. Román Mazuecos, M. A. Morillas Romerosa, M. Velasco Santos, I. D. De Juan, P. Vázquez Giraldo, H. Rincón Reques

**Affiliations:** 1 Servicio de Psiquiatría, Hospital Universitario La Paz; 2 Centro de Salud Mental de Tetuán, Madrid, Spain

## Abstract

**Introduction:**

Appearance and performance-enhancing drugs (APEDs) are a group of loosely-related substances that are used to improve characteristics perceived as desirable, such as external appearance, mental efficiency and sexual prowess. Besides the increasingly popular anabolic-androgenic steroids (AAS), there is an ever-growing array of anorectics, anabolism-enhancers, sedatives and nootropics available to the public.

**Objectives:**

This case report aims to explore the connection between emotional dysregulation, self-actualization and side-effects that arise in users of these drugs.

**Methods:**

We present the case of a 34-year-old male diagnosed with posttraumatic stress disorder and comorbid use of both anabolic-androgenic steroids (including testosterone, nandrolone and trenbolone) and chemsex-related substances (such as mephedrone, GHB and sildenafil). He reported frequent mood swings, constant feelings of loneliness and despair and occasional suicidal ideation; and attributed his pattern of substance abuse to a desire to escape from his “ordinary” self. After being admitted at a local emergency room during a suicidal crisis, he was referred to a mental health outpatient service and informed about the area’s addiction treatment resources. During the first outpatient interventions he related his symptoms (tachycardia, excessive sweating, restlessness, bursts of rage and paranoid reactions) to his pattern of APED consumption. He is currently undergoing both individual and group therapy in an MBT-based intervention; reports several months substance-free and has been started on a course of antidepressants (vortioxetine, up to 20 mg per day) and sedatives (zolpidem, 10 mg a day) to help him cope with his distress.

**Results:**

Several authors have explored the relationship between personality and APED use. While some have found a correlation between the “dark triad traits” (mostly machiavellianism) and AAS consumption, others have noted the role these substances play in building up both identity and self-worth. According to Macho et al. (Front Psychol 2021; 12:648467) APEDs provide access to an “actualized” and “extraordinary” self that enables amateur athletes to escape both everyday life and previous trauma. This way out might be dangerous for the user’s health, but proves to be an enticing road, particularly to young males such as the one we present in this case.

**Conclusions:**

APEDs are an increasingly popular group of substances, and they are going unnoticed in some clinical settings. Unlike other drugs, its use is not only recreational but directed to a specific goal; a quest for extraordinariness (in appearance, performance or prowess) that is deeply embedded in our culture. Having both a broad spectrum of physical side effects and a myriad of psychiatric implications, it is vital for mental health professionals to be familiar with this reality when assessing patients, particularly young men.

**Disclosure of Interest:**

None Declared

